# Exploring Nanoscale Structure in Perovskite Precursor Solutions Using Neutron and Light Scattering

**DOI:** 10.1021/acs.chemmater.2c00905

**Published:** 2022-08-03

**Authors:** Mary E. O’Kane, Joel A. Smith, Rachel C. Kilbride, Emma L. K. Spooner, Chris P. Duif, Thomas E. Catley, Adam L. Washington, Stephen M. King, Steven R. Parnell, Andrew J. Parnell

**Affiliations:** † Department of Physics and Astronomy, University of Sheffield, The Hicks Building, Sheffield S3 7RH, United Kingdom; ‡ Faculty of Applied Sciences, Delft University of Technology, Mekelweg 15, 2629 JB Delft, The Netherlands; § ISIS Pulsed Neutron and Muon Source, STFC Rutherford Appleton Laboratory, Harwell Campus, Didcot OX11 0QX, United Kingdom

## Abstract

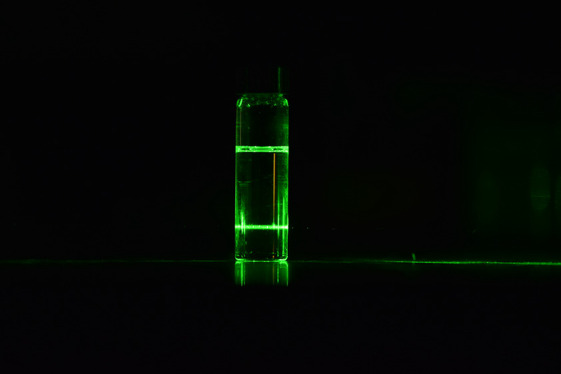

Tailoring the solution chemistry of metal halide perovskites requires a detailed understanding of precursor aggregation and coordination. In this work, we use various scattering techniques, including dynamic light scattering (DLS), small angle neutron scattering (SANS), and spin–echo SANS (SESANS) to probe the nanostructures from 1 nm to 10 μm within two different lead-halide perovskite solution inks (MAPbI_3_ and a triple-cation mixed-halide perovskite). We find that DLS can misrepresent the size distribution of the colloidal dispersion and use SANS/SESANS to confirm that these perovskite solutions are mostly comprised of 1–2 nm-sized particles. We further conclude that if there are larger colloids present, their concentration must be <0.005% of the total dispersion volume. With SANS, we apply a simple fitting model for two component microemulsions (Teubner–Strey), demonstrating this as a potential method to investigate the structure, chemical composition, and colloidal stability of perovskite solutions, and we here show that MAPbI_3_ solutions age more drastically than triple cation solutions.

## 1 Introduction

Halide perovskites have emerged as one of the most exciting new hybrid organic–inorganic photovoltaic material systems, capable of producing power conversion efficiencies of up to 25.7%^1^ due to several ideal material characteristics.^2−6^ A huge benefit of perovskite solar cells (PSCs) is that they can be processed at scale via low-cost solution processing techniques that are compatible with roll-to-roll manufacturing, such as spray coating, inkjet printing, and contact methods like slot-die, gravure, and blade coating.^7−10^ However, in order to achieve the highest quality perovskite films, every step of the crystallization process must be tightly controlled, from ink creation to device encapsulation. For these materials to be reliably cast from solution on an industrial scale, the structures and compositions formed within the precursor solutions, along with their usable lifetimes, must be more thoroughly understood. To do this requires characterization methods with suitable figures of merit that can relate a solution’s physical and chemical properties to their final material function in thin films. These insights could aid the rational design of new and improved perovskite precursor inks and the overall coating processes.

In 2015, Yan et al.^11^ used light scattering techniques to study methylammonium lead iodide (MAPbI_3_) precursor solutions. This work concluded that PSC precursors are not in fact “pure” solutions, in the sense that the precursors are not dissolved as ions or complexes. Instead, they suggested that these solutions were colloidal suspensions with colloids ranging in size from 10 to 1000 nm.^11^ Since this study, various scattering techniques and measurements have been used to investigate precursor solutions, including dynamic light scattering (DLS) and Tyndall scattering.^12−20^ Most of these studies revealed a bimodal size distribution within the precursor solutions. The bulk of PSC precursor solutions appear to be made up of ∼2 nm sized particles, with a second peak often seen at a much longer length scale, assigned to the presence of larger particles. However, the placement and intensity of this “larger colloid” peak varies significantly between solutions that are similar in composition, and there is still debate as to how much these colloids affect subsequent film structure formation and performance. For example, while some studies show the disaggregation of large colloids/aggregates leads to improved device performance,^18,19,21^ others show an optimum amount of large colloids results in the formation of a more stable precursor.^16^ In contrast, some studies using DLS were not able to measure particles >10 nm,^20^ and a study by Dutta et al.^22^ examining perovskite colloids using cryo-electron microscopy of MAPbI_3_ solutions found the dominant species to be small colloids. Consequently, it is reasonable to ask exactly what DLS is measuring in these solutions.

First, DLS or any form of light scattering from a polydisperse, or bimodal, system must always be interpreted with caution. For Rayleigh scattering, the intensity of the scattered light scales with *d*^6^, where *d* is the diameter of a particle^23^ (DLS theory is explored further in the Supporting Information). Thus, even a small number of large particles will dominate the measured signal in a way that is disproportionate to their volume fraction. This makes it inherently hard to study dispersions with a bimodal distribution. Additionally, with DLS there are potential complications that can arise from the inverse relationship between the derived size and the measured diffusion coefficient, because the intensity autocorrelation functions will decay at different rates over different ranges of delay times. The presence of unwanted contaminants such as dust particles can also impact results.

Small-angle neutron scattering (SANS) and small-angle X-ray scattering (SAXS) are powerful and proven techniques used for characterizing the size and shape of colloids in dispersion and macromolecules in solution^24−26^ and can readily cope with optically opaque samples, as the scattering results from periodic variations in nuclear scattering length and electron density, respectively. In 2021, Flatken et al.^27^ published a study examining perovskite precursor solutions using both SAXS and SANS, which enabled them to confirm the presence of particles at the nm length scale in MAPbI_3_ solutions. They modeled their small angle data using a cylindrical form factor of dimensions 0.5 nm × 0.7 nm (diameter × length), coupled with a hard sphere structure factor, that is, a lead-halide octahedra presenting as a cylinder. Their analysis also gave the spacing between the cylindrical particles as 2 nm. Given the limitations on the observable length scales with conventional SANS and SAXS measurements, DLS was used to confirm the presence of ∼2 μm colloids in solution.

Spin–echo SANS (SESANS) is an exciting neutron scattering development that combines the benefits of a neutron scattering technique with the ability to probe length scales from ∼100’s nm to 20 μm^28−30^ and, if necessary, also analyze highly concentrated systems as multiple scattering is accounted for.^31^ The SESANS technique “encodes” angular information arising from the scattering process in the precession of neutrons in a polarized beam (much like ^1^H NMR as neutrons are also spin-1/2 particles), thereby circumventing the strict geometrical limitations of conventional SANS/SAXS or ultrasmall-angle scattering (USANS/USAXS). SESANS has been previously used to study a number of soft matter systems,^29^ aggregates in solution,^28^ and colloidal particles.^30,32^ More recently Bernardo et al. used the technique to study the degree of dissolution of a range of organic photovoltaic relevant fullerenes (PC_61_BM and PC_71_BM) to ascertain the maximum solubility in these highly opaque solutions.^33^ They were able to observe the presence of colloidal aggregates in solution arising from incomplete dissolution of the fullerene species as they went beyond the solubility limit.

In this current work, we have investigated the aging of two common perovskite precursor systems: a triple cation (TC) perovskite precursor (Cs_0.05_FA_0.79_MA_0.16_Pb(I_0.85_Br_0.15_)_3_), where FA is formamidinium and MA is methylammonium, and a MAPbI_3_ solution, using both SANS and DLS to study the solution length scales. To cross-check these results and investigate further the presence of dispersed larger colloids, we have measured these same solutions using SESANS over a period of 9 days.

Through the combination of these three techniques, we are able to compare and contrast the length scales and size distributions of dispersed colloids present in perovskite precursor inks. Importantly, we conclude that there are only very small amounts of the large (μm-sized) colloids or equivalently sized contaminants. To assess the possible impact of the large colloids in thin films, we calculate a possible number of the micron-sized particles in a wet film, deducing that they are unlikely to act as nucleation sites and therefore are probably contaminants or defects.

By further analyzing the SANS data, we also reveal some interesting properties of the nm-sized colloids and observe that MAPbI_3_ solutions change drastically in chemical composition over a 3 month period, while the colloids dispersed within TC solutions remain relatively stable. This paper shows that the combined use of SANS and SESANS is a novel way of directly studying the structure of precursor perovskite species in solution across a broad length scale range, and as a proof of concept, we have shown that SANS could be a useful tool in further solution studies, and, in conjunction with other techniques such as NMR, we could obtain a more complete picture of how these precursors evolve over time.

## 2 Experimental Results

### 2.1 Precursor Solution and Film Preparation

All solvents were purchased from Sigma-Aldrich (Gillingham, UK). Pb-excess TC solutions, of 1.2 M:1.3 M and between 12 and 14 vol %, were made using the following concentrations: 507 mg/mL PbI_2_, 171 mg/mL FAI, 73.4 mg/mL PbBr_2_ ,and 22.3 mg/mL MABr in 4:1 DMF/DMSO with 50 μL of CsI added from a 1.5 M stock solution. MAPbI_3_ samples were made at 0.65 M and 8–9 vol % using the following concentrations: 300.8 mg/mL PbI_2_ and 103.8 mg/mL MAI in DMF. In both cases, these solutions were stirred overnight at room temperature to dissolve. Samples for absorption measurements were prepared on quartz-coated glass. All other characterization was conducted on ITO-coated glass. Glass substrates were purchased from Ossila.

All perovskite films were deposited in an inert environment. For MAPbI_3_ films, 50 μL of precursor was statically spin-coated using a two step process: 1000 rpm for 10 s, then 5000 rpm for 30 s. 100 μL of ethyl acetate was smoothly deposited onto the films 10 s from the end of deposition. For TC films, 35 μL of precursor was statically spin coated using a two step process: 1000 rpm for 10 s, then 6000 rpm for 20 s. 100 μL of chlorobenzene was deposited onto the films 5 s from the end of deposition. In both cases, the films were annealed at 100 °C for 20 min.

### 2.2 Dynamic Light Scattering

All the DLS measurements were acquired using a Zetasizer Nano-ZS (Malvern Panalytical, UK). Measurements were taken on the undiluted samples at 1.3 M for the TC solution and 0.65 M for the MAPbI_3_ solution, as has been the standard practice for previous DLS studies on perovskite precursor solutions in literature. The viscosity of the solvents used was calculated as ∼0.678 m.Pa·s. The absorption coefficients for lead and MAPbI_3_ are very similar for the wavelength used (633 nm), so the absorption coefficients for solutes in the solution were estimated to be 0.7. For each measurement, 10 scans each lasting 10 s were averaged to produce an intensity-weighted size distribution profile. This was repeated three times and the three scans averaged for each perovskite precursor ink solution. The intensity-weighted size distributions were then converted to volume-weighted size distributions using Mie theory (see Supplementary Note 1).

### 2.3 Small-Angle Neutron Scattering

The majority of SANS measurements were carried out on the LOQ diffractometer^34^ at the ISIS Spallation Neutron Source (STFC Rutherford Appleton Laboratory, Didcot, UK). LOQ is a fixed geometry, time-of-flight instrument that simultaneously records scattered neutrons with wavelengths of 2 ≤ λ ≤ 10 Å on two separate two-dimensional (2D) neutron detectors to provide a total *q*-range of ∼0.006–1.4 Å^–1^. For the solvent study on MAPbI_3_ precursors, SANS Xpress measurements were carried out on the beamline SANS2D at ISIS. For the SANS measurements, the perovskite precursor materials were prepared in deuterated DMF (DMF-*d*_7_) and DMSO (DMSO-*d*_6_) solvents to provide the necessary neutron scattering length density contrast and contained in 2 mm path length cylindrical quartz cuvettes (Hellma UK, Type 120; Starna Scientific Ltd., Type 32) which were mounted on a temperature-controlled sample changer. The solvent scattering length density (SLD) contrast for MAPbI_3_ was (ΔSLD = (SLD_solute_ – SLD_solvent_) ∼ 4.7 × 10^–6^ Å^–2^), while for the TC solution, ΔSLD ∼ 4.5 × 10^–6^ Å^–2^ (see Table S1). The incident neutron beam was collimated such that it was 10 mm in diameter at the sample position.

Each raw 2D scattering data set was corrected for the detector efficiency and spatial linearity and the measured neutron transmissions, before being azimuthally integrated and converted to the 1D coherent elastic scattering cross-section; herein referred to as the intensity, as a function of the scattering vector *q* (where *q* = 4π sin θ/λ, where 2θ is the scattering angle) using the Mantid framework (version 4.2.0). These data were then placed on an absolute scale (cm^–1^) by comparison with the scattering from a partially deuterated polystyrene blend of known molecular weight measured with the same instrument configuration. Background corrections arising from the quartz cuvette and the solvent were made in SasView version 4.2.2.^35^ The model fitting was also performed with SasView using the peak_lorentz and teubner_strey models. During aging, the solutions were stored in sealed, amber vials under ambient conditions.

### 2.4 Film Characterization

UV–vis measurements were performed under ambient conditions using a UV–vis/NIR light source (Ocean Optics, DH-2000-BAL), collection fiber optic cables (Ocean Optics), and spectrometer (Ocean Optics, HR2000+ES).

AFM measurements were obtained using a Veeco Dimension 3100 in intermittent contact (tapping) mode with a NuNano Scout 350 cantilever (nominal spring constant 42 N/m, resonant frequency 350 kHz). Each sample was scanned over three 10 × 10 μm areas. Data were analyzed using the open-source software Gwyddion^36^ to obtain roughness measurements. Height histograms were plotted, and all bearing analysis was completed using Nanoscope 1.9. SEM imaging was performed using an FEI Nova Nano450 SEM operating at a beam energy of 1.5 kV at a working distance of 4–5 mm, with an in-lens detector used to collect backscattered electrons. Grain sizes were processed using ImageJ software (version 1.52a). XRD measurements were performed using a PANalytical X’Pert^3^ Powder system. This was equipped with a Copper line X-ray tube operated at a voltage of 45 kV with a tube current of 40 mA, with data collected using a 1D detector, in Bragg–Brentano geometry.

### 2.5 Small Angle Spin–Echo Neutron Scattering

The SESANS dynamics measurements were undertaken at the Reactor Institute Delft (RID) at TU Delft, Netherlands. The instrument is described in Rekveldt et al.^37^ The solutions were again contained in cylindrical “banjo” quartz cuvettes (Hellma). These were mounted in a bespoke sample rotator to compensate for any sedimentation and to ensure that if there had been large “aggregates” or dispersed colloidal assemblies present, they would still have been measured using SESANS. The measured data were scaled relative to a nonscattering empty sample position; this is referred to as having the polarization *P*_0_, as there is no length scale (within the spin–echo length probed) able to depolarize the beam. This reference incorporates all the instrumental polarization as a function of spin–echo length. In a sample with length scales in the spin–echo range of the instrument, this results in a deviation from this reference state. Each of the perovskite solution SESANS measurements took 2 h, and measurements were regularly repeated up to a maximum of 9 days post-dissolution. The SESANS data presented are displayed as the ratio of the sample to the *P*_0_ divided by the wavelength squared. At RID, the neutron wavelength used is 2.06 Å. The colloidal silica comparison sample was measured on the LARMOR beamline at the ISIS Pulsed Neutron & Muon Source^38^ from 12 nm to 4 μm and at the RID up to 10 μm (the plotted data are a composite of both data sets). The sample was rotated at ∼3 rpm for both measurements to avoid sedimentation effects.

## 3 Results and Discussion

### 3.1 Dynamic Light Scattering

**Figure 1:**
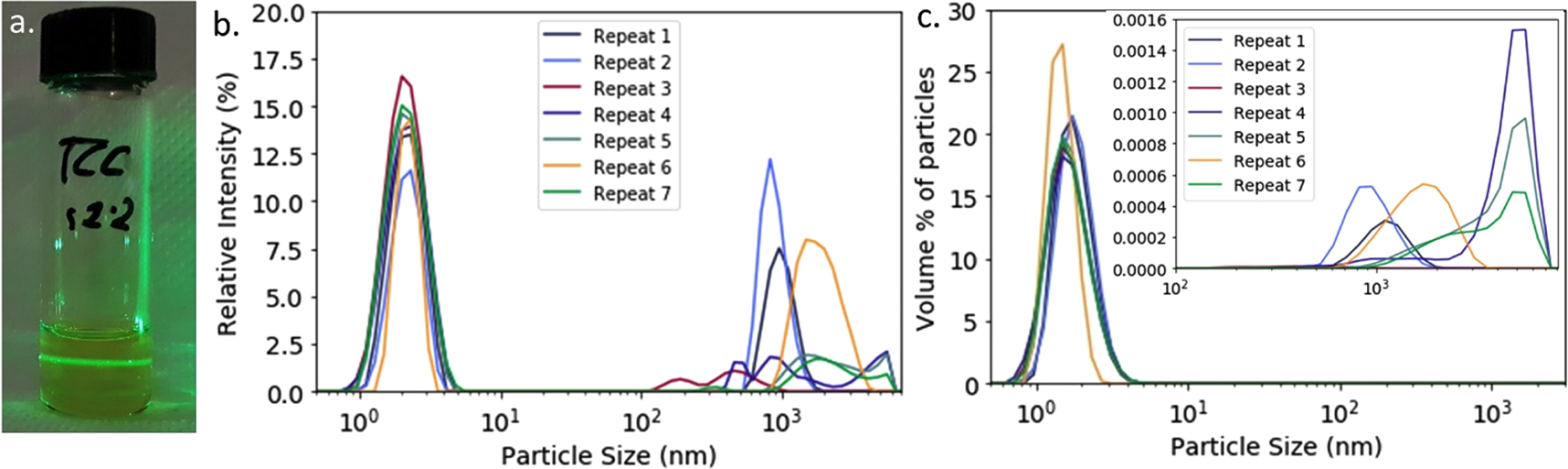
(a) Tyndall scattering from a TC perovskite solution, showing the presence of particles/colloids in suspension. The DLS size distribution data for seven distinct but compositionally identical TC solutions plotted in (b) intensity-weighted and (c) volume-weighted representations. The inset in (c) highlights the actual contribution from the larger colloids/particles.

Figure 1a clearly shows the path of a green laser through a ∼30 wt % TC solution. In accordance with Tyndall scattering, this proves that this perovskite precursor is a colloidal suspension. However, we cannot deduce the particle size or concentration in solution from this simple qualitative observation. Figure 1b shows the DLS size distributions for seven distinct TC solutions with identical chemical composition, labeled repeat 1, 2, and 3, etc., plotted in terms of intensity. All three solutions give a bimodal distribution, consistently centered around ∼1–3 nm and with a secondary population at 100–8000 nm. Given that they have the same chemical composition and stoichiometry, these solutions should have the same or very similar populations of particles when measured using this technique. However, repeats 1, 2, and 6 have a much greater proportion of larger colloids than were measured for other solutions. This may be due to differences in dissolution of the material related to the initial size of the dry material powder. We notice a wide variation in both peak shape and location for these larger colloids. In fact, most solutions show multiple peaks at this length scale. To investigate this further, we look more stringently at the data. Each measurement shown in Figure 1 is averaged over three individual measurements taken successively. If we examine the individual measurements (as shown in Figure S2), we see the placement of these peaks changes even over the course of the measurement. This gives us cause to believe these “large colloids” are diffusing in and out of the incident light pathway, leading to these very varied measurements. We believe therefore that these particles are quite sparse in the solution. Additionally, Figure S3 shows that these larger peaks disappear after filtration, indicating that this is not a measurement artifact. We discuss this further in the Supporting Information, as small amounts of large particles can distort the DLS intensity-weighted size distribution.^23,39^ In order to further understand the true significance of this bimodal distribution, the DLS data must be volume weighted to give an accurate representation of colloid size by volume fraction. Volume percentage distributions of the solutions are also shown in Figure 1c. We see that the peaks in Figure 1b derive from a near negligible volume percentage of larger colloids.

It has been suggested that these μm-sized colloids can influence the subsequent perovskite thin-film grain size, by acting as nucleation sites for the perovskite phase during crystallization.^21^ We have investigated this idea with calculations based on our DLS measurements (calculation specifics are detailed in Supporting Information). Using SEM images of annealed perovskite films made with TC solutions, we find the average grain density and compare this to the number of particles in solution. We assume that to spin coat a perovskite film requires an initial wet film thickness of ∼10 μm. We approximate a total solute concentration of ∼14 vol % within our TC solutions and assume the particles are spherical. We used the solution with the highest volume fraction of larger particles (repeat 1). According to our DLS data, 0.000024 vol % of the solutes in this solution have a diameter of ∼920 nm and the other 99.999976% of these particles have a diameter of 1.6 nm. If this is the case, then in 1 mL of the TC solution, there are ∼8 × 10^6^ μm-sized particles, compared to ∼5.4 × 10^19^ nm-sized particles. If these particles are distributed uniformly throughout all precursor material, a wet film with an area of 1 cm^2^ and thickness of 10 μm would therefore contain ∼8000 large particles and ∼5.8 × 10^16^ small particles.

**Figure 2:**
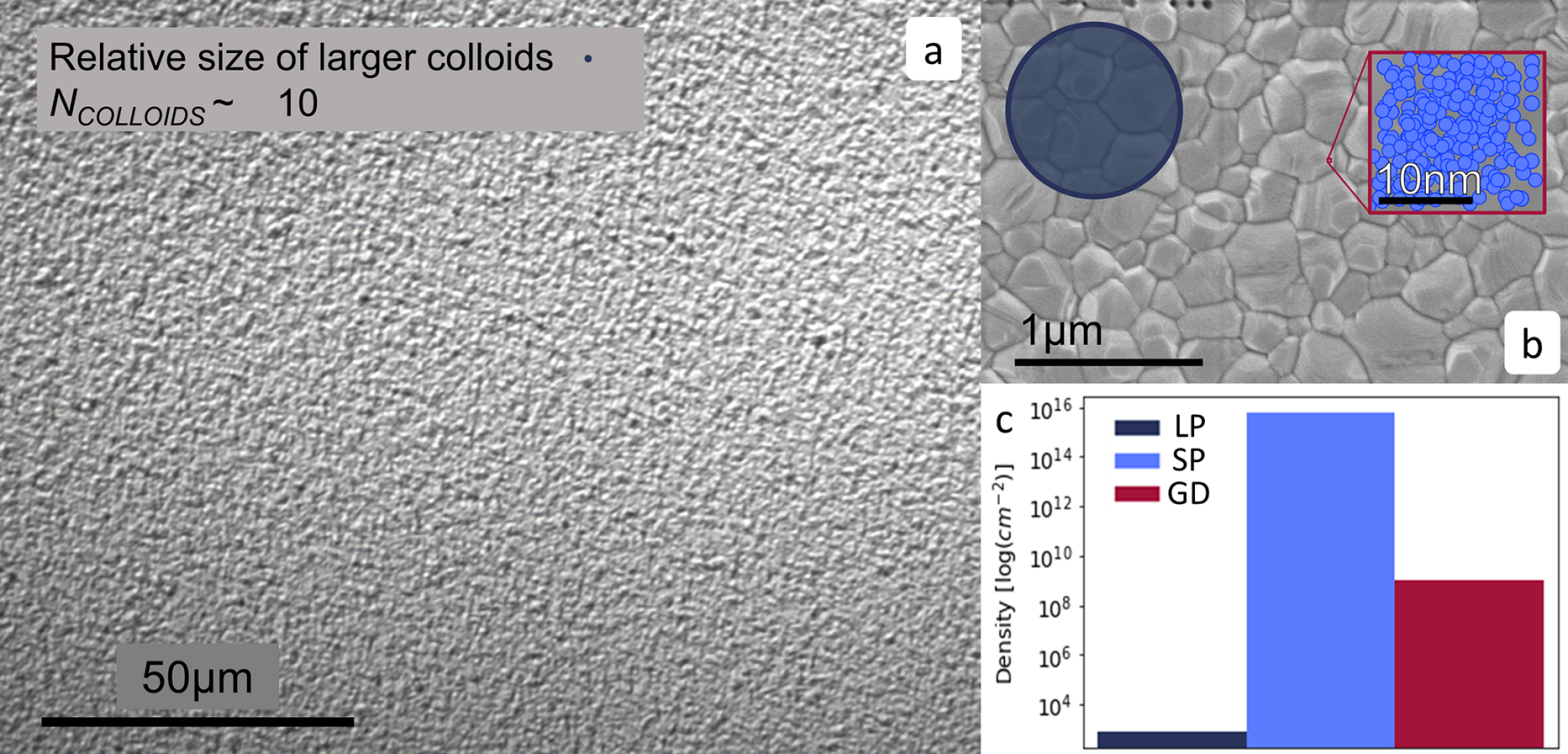
An optical microscope image (a) and a SEM image (b) of a thermally annealed TC perovskite film with an average grain size of ∼200–600 nm. For both images, a micron-sized particle is shown for reference (dark blue). (b) The inset contains a magnified area of this image along a schematic illustration showing the relative size and density of the smaller particles (light blue). (c) Estimated number density of large and smaller particles dispersions in a wet film of 10 μm thickness (LP and SP, respectively), compared to the average grain density (GD) in the final annealed perovskite film; the data is plotted on a log scale.

Figure 2 explores what these numbers mean physically in an annealed perovskite film. Figure 2a shows an optical image of a perovskite film at 50× magnification. According to our calculation, there would be ∼10 large colloids in this area. Figure 2b shows an example of an SEM image of a TC film. Here, we see that neither the large nor the small particles sizes align with grains in the final perovskite film in either density or scale. Given the size and distribution of these large colloids, they are more likely to appear as defects than to enhance film crystallization and are too few in number to be responsible for the observed perovskite grain size. We therefore postulate that these grains must instead result from nucleation from the dominant population of smaller 1.7 nm particles.

### 3.2 Neutron Scattering: SANS

To further investigate the dispersed structures in solution, we conducted SANS and SESANS measurements on both the TC precursor (molarity 1.3 M) and a MAPbI_3_ precursor solution (0.65 M). To provide suitable neutron contrast between the bulk medium and PbI_2_, we used deuterated solvents, *d*_7_-DMF and *d*_6_-DMSO (see Table S1). This has the added benefit of reducing the incoherent background scattering level, thereby improving the signal-to-noise ratio. This is important because the molar concentrations of our precursor solutions constitute very dilute systems in volume fraction terms and means the SANS intensities are correspondingly low. However, the contrast in our systems is better than that in a silica/D_2_O dispersion, a colloidal system that has been widely studied with SANS.^40^ Thus, we can have high confidence that our SANS/SESANS measurements will be sensitive to lead-based dispersed colloids or aggregates at low concentrations if they are present in our samples.

**Figure 3:**
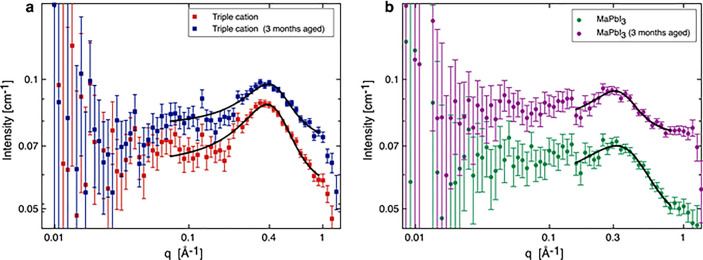
SANS data for (a) TC and (b) MAPbI_3_ perovskite precursor solutions fitted with a Lorentzian peak model (black line). The data in each plot compare the 3 month-aged solutions that correspond to the samples measured by SESANS and fresh solutions measured soon after dissolution.

To explore if the SANS technique could map changes in the perovskite solutions, we measured two different perovskite precursors (TC and MAPbI_3_) before and after 3 months of solution aging. The results are shown in Figure 3. As anticipated, the absolute scattering intensities are very small with a concomitant degree of statistical uncertainty, particularly at smaller scattering vectors where there are relatively fewer neutrons. Nevertheless, at larger scattering vectors, each set of data clearly shows a broad peak. These peaks were initially fitted to a Lorentzian function to ascertain their position. The TC system (in Figure 3a) has peaks positioned at *q* values of 0.394 Å^–1^ for the aged sample and 0.385 Å^–1^ the fresh sample, corresponding to length scales (= 2π/*q*_peak_) of 1.59 and 1.63 nm, respectively. These length scales reassuringly agree with the DLS results attained earlier, which give a particle size of 1.6 nm for the TC system. For the MAPbI_3_ solutions, the same analysis gave peak *q* values at 0.315 Å^–1^ for the fresh repeat and 0.308 Å^–1^ for the aged sample. These smaller *q* values correspond to slightly *larger* length scales of 1.99 nm for the repeat and 2.04 nm for the aged sample, respectively.

The broad nature of the peaks indicates that there is no regular repeating structure or single fixed distance. Furthermore, the absence of any power law regions in the data at low *q* indicates an absence of both mass fractal (e.g., cluster aggregation gives intensity ∼ *q*^–2^) and surface fractal (yielding *q*^–3^ – *q*^–4^ depending on the surface roughness) behavior within the SANS measurement range. The latter is indirect corroboration of the DLS findings; namely, that if any micron-sized aggregates dispersed in solution are present, they are so few as to have a negligible impact on the SANS.

In order to reduce the number of assumptions made about the structure of these nanoparticles, we have taken a simple approach to modeling this, employing the model developed by Teubner and Strey.^41,42^ The Teubner–Strey formalism models the scattering of an interacting two-phase system *without long-range order* characterized by a correlation length, ξ, the length scale beyond which correlations die out, and domain size/periodicity, *d*. This is discussed further in the Supporting Information. Below is the formula used to fit the SANS data (eqs 1–4):^41^
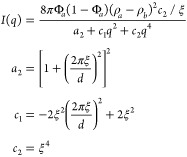
where Φ_*a*_ represents the volume fraction of one of the phases, and (ρ_*a*_ – ρ_*b*_) is the contrast in scattering length densities between these two phases. The Teubner–Strey model has been widely used to describe micellar and microemulsion systems. Indeed, we note that our perovskite precursor solutions satisfy the same criteria outlined by Teubner and Strey for microemulsions, *a*_2_ > 0, *c*_1_ < 0, and *c*_2_ > 0,^41,42^ which provides some reassurance that the use of this model is appropriate.

**Figure 4:**
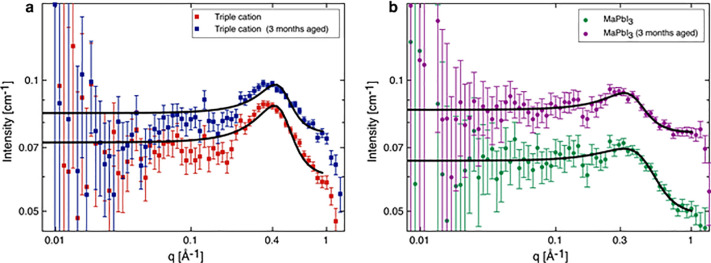
SANS data for (a) TC and (b) MAPbI_3_ perovskite precursor solutions. The data in each plot compare the 3 month-aged solutions (corresponding to samples measured for SESANS) and fresh solutions measured soon after dissolution. The continuous lines are fits with the Teubner–Strey model.

In applying the Teubner–Strey model shown in Figure 4, we constrain parameter space by assuming that the perovskite precursor solutions are characterized by two volume fractions, one consisting of primarily DMF/DMSO or DMF, and the other being richer in solute, containing lead halide complexes, organic cations, and possibly some solvent molecules. The SLDs of these two phases are represented by ρ_*a*_ and ρ_*b*_, respectively, while Φ_*a*_ represents the volume fraction of the solution. The Teubner–Strey model fits to our SANS data are shown in Figure 4, and the model parameters can be found in Table 1. During model-fitting, the neutron SLD of the bulk solvent phase was fixed at its calculated value based on its composition; all other parameters were allowed to optimize.

**Table 1:** Teubner–Strey Model Parameters Derived from Fits to the SANS Data for CsFAMAPb(Br_*x*_I_*x*–1_)_3_ in *d*_7_-DMF:*d*_6_-DMSO Solvent Blend and MAPI_3_ Perovskite Precursor Solutions Dissolved in Pure *d*_7_-DMF Shown in Figure 4^a^

sample	perovskite volume fraction (solute phase)	perovskite (solute phase) SLD (10^–6^ Å^–2^)	correlation length, ξ (nm)	domain size, *d* (nm)
triple cation (fresh)	0.140 ± 0.007	2.66 ± 0.07	0.59 ± 0.03	1.41 ± 0.02
triple cation (aged)	0.121 ± 0.007	2.89 ± 0.19	0.64 ± 0.05	1.40 ± 0.02
MAPbI_3_ (fresh)	0.059 ± 0.005	1.51 ± 0.18	0.41 ± 0.03	1.56 ± 0.03
MAPbI_3_ (aged)	0.078 ± 0.006	3.53 ± 0.10	0.63 ± 0.05	1.76 ± 0.04

a The SLD of the solvent bulk phase was fixed at its calculated value; this was 6.3 × 10^–6^ Å^–2^ for MAPbI_3_ and 6.12 × 10^–6^ Å^–2^ for the TC solutions.

The domain sizes compare favorably with our own DLS data and agree well with other SANS measurements from the literature.^27^ However, the simplicity of this model allows the SLD of this “solute domain” to be optimized and the composition determined, and this has provided us with an intriguing insight into the structure of these precursor solutions. Apart from the fresh MAPbI_3_ sample, the SLD values for the perovskite components are found to be *higher* than the calculated values for the pure phases or their mixtures likely to be present (see Table S1). There are several possible explanations for this: First, our SLD calculations have underestimated the bulk densities of the different components, although variations in material density will only give relatively small and linearly proportional changes in the SLD. Second, the solute-rich domains have coordinated/entrained (deuterated) solvent molecules. We consider the latter of these to be more likely.

In the MAPbI_3_ solution, both the size and SLD (composition) of the solute phase change substantially over the ∼3 months aging period. These changes in the solution structure suggest that the solute-rich domains we observe are metastable states, which can change in size or composition over time. This aligns with previous work that has identified the acidity, solvent decomposition, and additives can all affect perovskite solution structures.^12,19,21^ This increase in the domain size and correlation length implies that over time these nm-sized aggregates are growing.

In contrast, we can see that after 3 months of aging, neither the correlation length nor the domain size have substantially changed in the TC system. But, once again, the SLD of the solute-rich phase giving rise to the scattering does not correspond to that of any of the individual components shown in Table S1. This again suggests that there must be one or more intermediate complexes forming within the solutions with dimensions of ∼1.3–1.4 nm. This size value also correlates well with the DLS results.

There are many differences between these two systems that could result in these different aging processes (this includes solvent blend, molarity and solids composition). Based on what we have found previously,^43^ we speculate that deprotonated methylammonium (MA^+^) is very unstable in TC solutions. We hereby note that additional molecules in the TC sample (formamidinium, bromine, cesium) could potentially have a stabilizing effect on some of the intermediate complexes formed.^100^ We also note that in this study, the TC solution is dissolved in a DMF:DMSO solvent blend, while the MAPbI_3_ sample is dissolved purely in DMF. It has been shown that solvent coordination can affect the crystallization rate for perovskite films, for example, DMSO can form strong intermediate phases with different iodoplumbate structures^44^ during crystallization,^45−51^ and this stronger coordination can reduce some preliminary Pb–I coordinating and bridging interactions.^49,52^ Initially we suspected that this reduced solvent coordination causes the solute domains in the MAPbI_3_ sample to be more dynamic over time. To explore this idea further, we aged the MAPbI_3_ solutions in a DMF/DMSO solvent blend as with the TC solutions and detected the change in colloid populations using SANS. The results are shown in Figure S15, and we again find flat scattering at low *q*; however, the Teubner–Strey fits give unphysical volume fractions which may mean that they are not simple two phase solutions. This demonstrates that solvent choice plays an interesting role in the formation of these dispersions. We simply conclude here that these systems are much more complex than we originally suspected. However, we remark that SANS is a valuable tool to compare the composition of these different solutions, and this should be the subject of future studies.

We have demonstrated that these small particles are unlikely to be pure Pb-halide aggregates and appear to contain some entrained/complexed solvent molecules. By studying these two different systems, we have highlighted that SANS can be a useful tool to explore the size and, using a simple model, the composition of these nm-sized dispersions. We have also shown that in this instance, precursor dispersions are more stable for the TC-based solutions than for MAPbI_3_.

### 3.3 Film Morphology and Composition

We will now explore what effect these evolving dispersions have on the resulting perovskite films. Figure S7 show that the absorption spectra and the positions of XRD peaks for these films do not shift as the solutions age, indicating that there is little change to the perovskite film composition over this time. The only remarkable change is in the TC films, in the reduction of the PbI_2_ peak at 12.8° over time. This is expected, as there is an excess of Pb in these solutions. Figures S8 and S11 show SEM and AFM images of films made from different solutions for MAPbI_3_ and TC precursors, respectively. We see here that the MAPbI_3_ films have extremely different structures compared to TC films, as expected as they are such different systems. This MAPbI_3_ structure is explored in Figures S10 and S11, which contain height histograms and bearing analysis data taken from aged and fresh MAPbI_3_ AFM images.

In Figure S8, we can see MAPbI_3_ films show a characteristic “plank” structure that has been seen before, where DMF is the lone solvent for MAPbI_3_ perovskites.^11,53^ We find as these solutions age, the surface morphology of the subsequent film changes. Figure S8a,b shows the films from fresh solutions contain very broad structures, although there are significant pinholes. Figure S8a also shows striking bright spots, which could imply significant charge build-up within these pinholes. Figure S8b shows that all of these plank-like features are at a similar height, and we see a clear grain structure can be detected within them. In Figure S9, the height histogram for fresh MAPbI_3_ shows a single height average at around 400 nm, which also suggests a uniform height profile.

Figure S8c,d shows some interesting changes to this morphology. These structures are still present after ink aging; however, they appear to be a lot more disordered. We can see in Figure S8d, these fragmented structures lead to a much more inhomogeneous film. This observation is supported by the roughnesses measurements taken from this AFM data. The films made from aged MAPbI_3_ solutions are significantly rougher than their fresh counterparts; we measured RMS roughness of 137 ± 4 nm and 91 ± 8 nm for aged and fresh MAPbI_3_ respectively. Additionally, this is confirmed by the height histograms in Figure S9, which show a much broader peak for the aged ink than for fresh MAPbI_3_ samples. To probe this in more detail, we analyzed the bearing data for these height histograms. By integrating the area under these histograms, we can find what percentage of the overall film lies at various heights from the top surface of the film (Figure S10a). Figure S10b shows that for both films, the bulk of perovskite material lies between 300 and 700 nm. However, while the sample from a fresh solution shows a steep change in the curve at around 400 nm, the aged MAPbI_3_ sample shows a shallower gradient between 300 and 700 nm. This indicates the features in the film made from aged MAPbI_3_ solutions are arranged at random heights, leading to a much less orderly film. We believe this all indicates that there is an altered crystallization pathway for the film made from aged MAPbI_3_ solution compared to the fresh MAPbI_3_ solutions, and this change is linked to the larger precursor dispersions seen in Table 1.

To substantiate this, we refer to the SEM and AFM for the TC films. In Figure S11, we can see that grain size is significantly smaller after solution aging, but otherwise, the structure of the perovskite film remains largely the same and produces a dense, uniform film. To confirm these results, we have measured the roughness for these films and found there was no significant difference between the two films, 17.5 ± 0.7 nm and 15.8 ± 0.4 nm for fresh and aged TC, respectively.

We therefore believe these precursor dispersions affect perovskite formation pathways, hence the change in MAPbI_3_ precursors correlates with a change in film morphology. However, we acknowledge that more work should be done to understand how this will impact devices with more long-term aging. We also realize there are numerous other factors that are at play here and further study should include a more comprehensive sample selection, alongside a more comprehensive device study.

### 3.4 Neutron Scattering: SESANS

**Figure 5:**
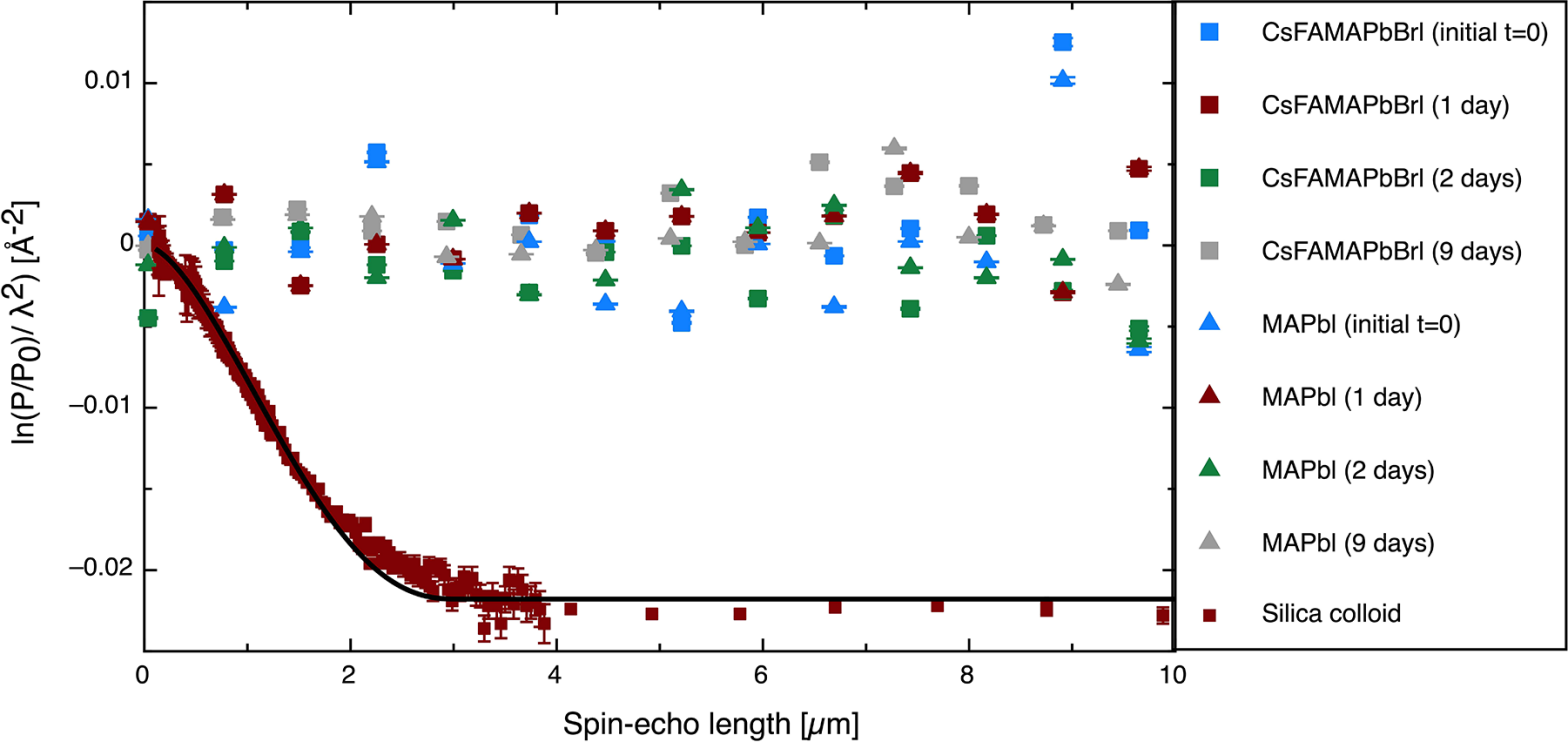
SESANS data for the TC (labeled as CsFAMAPbBrI) and MAPbI_3_ precursor solutions (labeled as MAPbI) measured at various times as indicated in the legend, from the point after dissolution (*t* = 0) and up to 9 days after dissolution. For comparison, a SESANS measurement of silica spheres in D_2_O showing scattering up to a length-scale characteristic of the particle diameter 3 μm is also shown; this was 0.1% by volume.

SESANS uses a polarized beam of neutrons traveling through a series of magnetic fields. By measuring the polarization of scattered neutrons which have interacted with the sample relative to those from the unscattered beam, we can measure the volume fraction of larger colloids in a solution (this is discussed in detail in Supporting Information). The SESANS data for the two distinct perovskite compositions are shown in Figure 5. These data were acquired continuously over a long period of time, from the initial dissolution of the perovskite powder precursor for a total of 9 days, with the solutions being measured at intervals throughout this period. To avoid sedimentation of these larger aggregates, these solutions were slowly rotated (∼3 rpm) as they were measured. While we acknowledge there is a chance that this rotation could break up the larger colloids dispersion, we reason that this disturbance would be no more violent than moving a solution from vial to cuvette before DLS measurements or before perovskite deposition. Additionally, as these solutions are measured over a long period, if these aggregates are going to form, they probably would form to some degree in these conditions, even with minor disturbances. These measurements should be sensitive to a concentration above 0.005 vol % for particles of radius 500 nm and 0.001 vol % for particles of radius 1 μm.^33^ The SESANS measurements unequivocally support the volume-weighted DLS results in that they both show a very low or effectively zero volume fraction of dispersed lead-based colloids present at larger particle sizes. To validate our approach and to illustrate what would be seen if such dispersed particles were present in solution, we have included SESANS data for a 3 μm diameter silica particle dispersion in D_2_O, this was at 0.1% by volume. Here, we can see a signal with a sharp drop in the polarization and a constant total scattering value at spin–echo length of ∼2–10 μm.

## 4 Conclusions

Within this paper, we have come to three main conclusions. The first is that DLS alone should be used cautiously when analyzing particle sizes within perovskite precursor solutions. While there is reliable and useful information in the “small particle” data, the intensity of light scattering from the large particles can lead to misinterpretations of the quantity of “large particles” present. It is therefore important that the volume-weighted size distributions are studied, in addition to the intensity-weighted size distributions routinely provided by DLS measurements if a true representation of the size distribution in these precursor solutions is to be obtained. We have also outlined the physical significance of these particle sizes and their role in the context of an annealed perovskite film and shown that if these larger particles are there (making up <0.0001% of the solution volume), it is unlikely that they have a beneficial effect on grain sizes or film quality; more likely introducing inhomogeneities or defects in the film.

We have also shown that both SANS and SESANS can be valuable tools when probing dispersed colloidal material within perovskite solutions. Using both techniques, we can span the length scales of 1 nm to 10 μm. By applying the Teubner–Strey model to our SANS data and fixing the known SLD of the majority solvent phase, we have revealed that these nm-sized particles are not uniquely Pb-halides, but more likely perovskite-solvent complexes. Further experiments should help illuminate how these components interact within these complex systems. Indeed, it may be possible to use a whole series of SLD solution contrasts to unambiguously determine the composition of these species. Additionally, SESANS has confirmed that there are vanishingly small amounts of the dispersed large colloids, being below the level of detection within either of the precursor solutions studied, agreeing with the conclusions found from DLS. We believe that both these techniques could, in conjunction with other techniques such as NMR, help us fully understand these precursor dispersions.

Lastly, through the aging of two perovskite solutions (TC and MAPbI_3_), we have examined solution chemistry over long time periods (3 months). The size and SLD of the nm-sized particles within the aged MAPbI_3_ sample are significantly different to the fresh MAPbI_3_ sample, whereas the TC sample stayed relatively consistent. We have related this to a change in surface morphology for the subsequent films. While there are many factors which could contribute to this observation, we note that the crystallization formation dynamics are altered for films made from aged MAPbI_3_ and we think relates to changes in precursor solutions seen with SANS. Exploring this relationship further should be the aim of future studies.

## Supporting Information Available

Additional experimental theory regarding the various scattering techniques used, additional SESANS data, and the scattering length densities of the various components studied (PDF)

## Abbreviations

TC: triple cation
DLS: dynamic light scattering
SANS: small-angle neutron scattering
SESANS: spin–echo small-angle neutron scattering
Cs: cesium
FA: formamidinium
MA: methylammonium
SAXS: small-angle X-ray scattering
