# Supplementary material for: Exploring Nanoscale Structure in Perovskite Precursor Solutions Using Neutron and Light Scattering
Source: Chem Mater. 2022 Aug 3;34(16):7232–41. doi: 10.1021/acs.chemmater.2c00905 (PMC9404541; doi:10.1021/acs.chemmater.2c00905)
Supplement: Supplementary file 1 — cm2c00905_si_001.pdf [file cm2c00905_si_001.pdf]

Supplementary Information

Exploring Nanoscale Structure in Perovskite Precursor Solutions Using Neutron and Light Scattering

Mary E. O'Kane[1], Joel A. Smith[1], Rachel C. Kilbride[1], Emma L. K. Spooner[1], Chris P. Duif[2], Thomas E. Catley[1], Adam L. Washington[3], Stephen M. King[3], Steven R. Parnell[2*] and Andrew J. Parnell[1*]

corresponding authors* , s.r.parnell@tudelft.nl, a.j.parnell@sheffield.ac.uk

[1]Department of Physics and Astronomy, University of Sheffield, The Hicks Building, Sheffield, S3 7RH., [2]Faculty of Applied Sciences, Delft University of Technology, Mekelweg 15, 2629 JB, Delft, The Netherlands. [3]ISIS Pulsed Neutron & Muon Source, STFC Rutherford Appleton Laboratory, Harwell Campus, Didcot, OX11 0QX

Supplementary Figure 1: Schematic of Dynamic Light Scattering Technique ……………………..2

Supplementary Figure 2: Intensity-weighted DLS over the course of a measurement……………. 4

Supplementary Figure 3: Intensity-weighted DLS for Filtered vs. Unfiltered PSC solution ……....5

Supplementary Figure 4: Schematics to Accompany DLS Calculations…………………………...6

Supplementary Figure 5: Schematic of Small-Angle Neutron Scattering Technique………………7

Supplementary Figure 6: Illustration of Physical Meaning of Teubner-Strey Model in PSC inks…8

Supplementary Figure 7: Absorption and XRD patterns of films from aging inks………………...10

Supplementary Figure 8: SEM/AFM images of fresh and aged $MAPbI_3$…………………………11

Supplementary Figure 9: Height histograms for $MAPbI_3$ films ……….………………………...12

Supplementary Figure 10: Bearing analysis data for $MAPbI_3$ films ………………………………13

Supplementary Figure 11: SEM/AFM images of fresh and aged TC ……………………………..14

Supplementary Figure 12: Schematic of Spin-Echo SANS Technique……………………………14

Supplementary Figure 13: SESANS data for Triple Cation Perovskite Precursors..................16

Supplementary Figure 14: SESANS data for $MAPbI_3$ Perovskite Precursors........................16

Supplementary Figure 15: SANS of $MAPbI_3$ Perovskite Precursors (DMF/DMSO)...............17

Dynamic Light Scattering

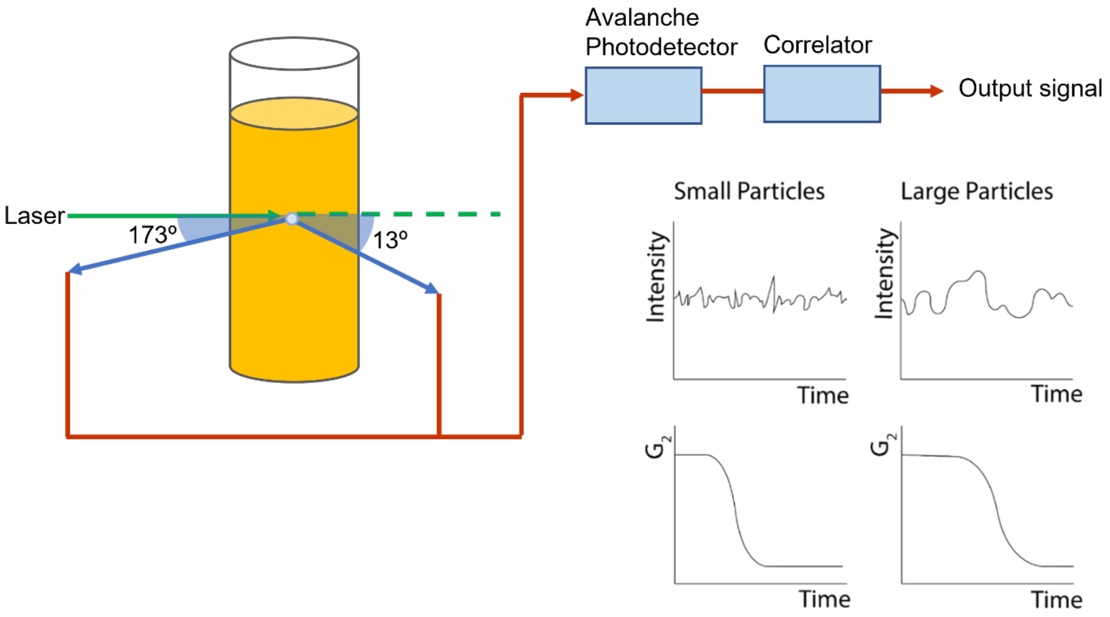

Figure S1. Schematic depicting dynamic light scattering from a Zetasizer Nano ZS DLS machine, adapted from source[1]

In conventional dynamic light scattering, as used in this study, a laser is directed into the colloidal suspension and the intensity of the scattered light is detected at a set angle. This will contain a combination of constructive and destructive interferences as the light randomly scatters off colloids in the dispersion. This interference pattern changes in intensity as the particles move due to Brownian motion which in turn depends on the particle size as well as the temperature and viscosity of the solvent. Larger particles diffuse more slowly.[2,3] DLS measures the fluctuations in the interference pattern over time, this process is shown schematically in Figure S1. The output of the measurement is an autocorrelation function showing the fluctuation of intensity over time called G_2 [1]. G_2 can be related to the electric field autocorrelation function G_1 which measures the averages motion of particles relative to one another.

$$G_2(\tau) = B + \beta|G_1(\tau)|^2 \qquad \text{Equation S1}$$

Where B is a baseline constant, τ is the lag time between time (*t*) and (*t*+ *τ*) and β is factor associated with the measuring equipment. For a polydisperse system, G_1 can be written as:

$$G_1 = \int_0^{\infty} G(\Gamma)e^{-\Gamma_\tau dt} \qquad \text{Equation S2}$$

Where

$$\Gamma = -D_\tau q^2 \qquad \text{Equation S3}$$

Here, Γ is a decay constant that is directly related to the diffusion constant of the colloids in dispersion over time increment D_τ. Whereas, q is the scattering vector (which incorporates the refractive index of the solvent[2]). The hydrodynamic radius R_h of a molecule can be determined through the Stokes-Einstein equation

$$D_\tau = \frac{k_B T}{6\pi\eta R_h} \qquad \text{Equation S4}$$

Where T is temperature at which the measurements are taken, k_B is the Boltzmann constant and η is the viscosity of the solvent. Through the above equations, a series of exponential decays can be fitted to G_2 for each particle population to determine R_h. As can be seen in Figure S1, the exponential decay G_2 will take longer for larger colloids.[3] Additionally, the more extended the decay becomes, the greater the polydispersity of the colloids in the dispersion.

For particles much smaller than the probe lasers wavelength (d< λ/10), light is scattered uniformly according to Rayleigh scattering theory. However, for particles comparable in size to λ the scattering can be described by Mie scattering theory, which depends on the refractive index of the scattering colloid and solvent, as well as the shape of the colloid, resulting in a pronounced angular dependence to the scattering [2].

The intensity of Rayleigh scattering (I) is proportional to the square of the volume of the colloid and thus to its radius (r) as

$$I \propto r^6 \quad \text{Equation S5}$$

This means that a particle with r = 50 nm will scatter with a million times the intensity of a particle with a radius of r = 5 nm. This is a serious consideration when studying multimodal particle dispersions, as the scattering from a few larger particles will dominate that from many more but smaller particles. Mie theory can be used to convert data from this intensity-weighted domain into either a number-weighted or, as here, a volume-weighted domain.

For a solution containing N_a particles of size *a,* and N_b particles of size *b,* the magnitude of the intensity peak at size a is evaluated using:

$$\%\, I_a = 100 \times \frac{a^6 N_a}{a^6 N_a + b^6 N_b} \qquad \text{Equation S6}$$

Using the Rayleigh approximation, in which mass is proportional to r^3- the volume percentage of colloid a can be estimated using:

$$\% V_a = 100 \times \frac{a^3 N_a}{a^3 N_a + b^3 N_b} \qquad \text{Equation S7}$$

In this work, spherical geometry is assumed for the conversion from intensity to volume.

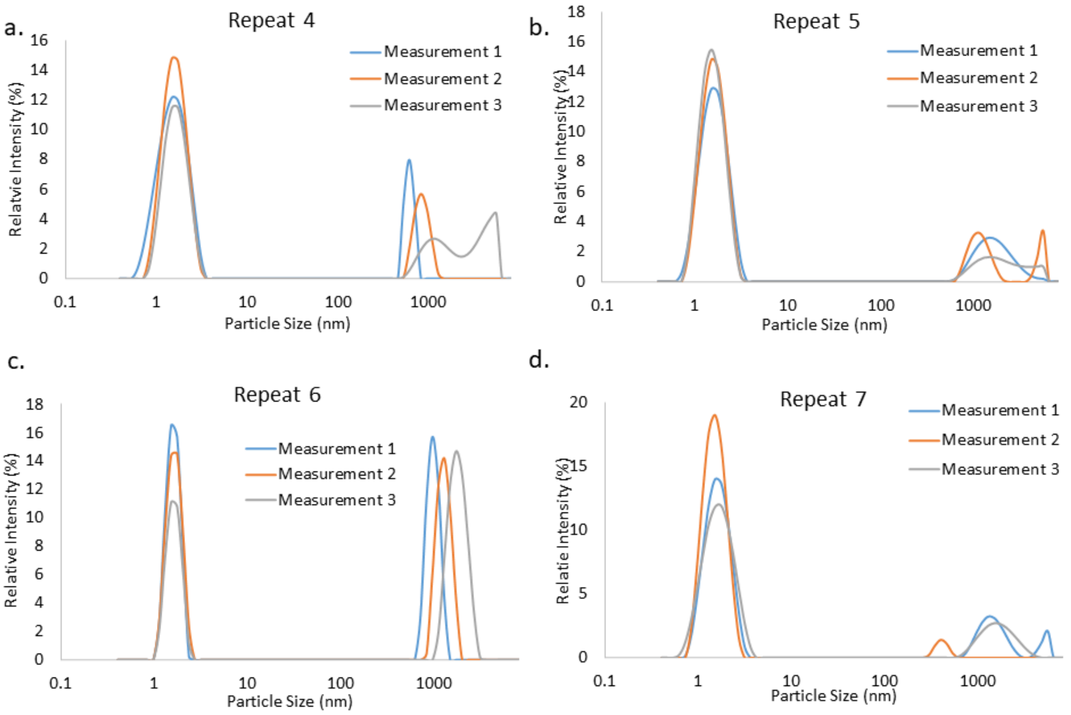

Figure S2. Individual Intensity-weighted DLS size distribution graphs for Repeat 4 **(a)**, 5 **(b)**, 6 **(c)** and 7 **(d)**. Three intensity profiles are taken over the course of a measurement (Measurements 1,2 and 3) and these are averaged to provide the results in Figure 1a.

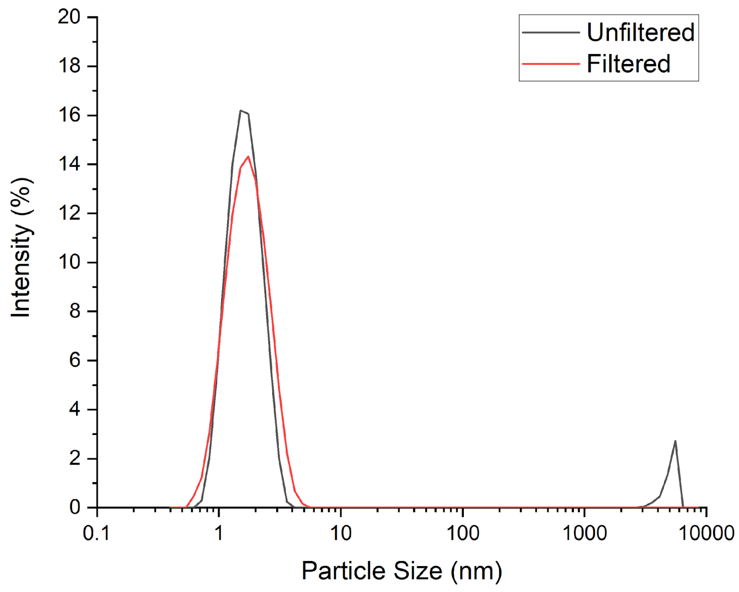

Figure S3. Intensity-weighted DLS size distributions from unfiltered (black line) and filtered through a 0.2 µm filter (red line) fresh TC perovskite precursor solutions at 1.3M.

Film Calculations

From the volume-weighted DLS size distributions, we assume a Gaussian distribution around a mean particle size and use the area underneath this curve to find the Vol% of a particular particle size as shown in Figure S3.

These data represent the hydrodynamic diameter of these particles therefore the particle radius is half this. If we assume these are spherical colloids, then the volume of a large and small particle is $4.1\text{x}10^{-13}$ cm^{-3} and $2.6\text{x}10^{-21}$ cm^{-3}, respectively. The total solute vol% of these TC solutions is roughly 14% and from the DLS we can tell that of this 0.0000235% is made up of large particles. Therefore, in any volume of TC solution, $3.291\text{x}10^{-6}$ % of that will made up of large particles. So given the volume of a particle and how much volume they all occupy, we can estimate how many there are. Following through, we find that there are $8.11\text{x}10^{6}$ large particles in 1ml. In a wet film of average thickness 10µm, this would lead to 8150 large particles/cm^2. By the same logic, we calculate that there are $5.4\text{x}10^{19}$ small particles in 1ml and therefore $5.78\text{x}10^{16}$ small particles/cm^2 in the same wet film.

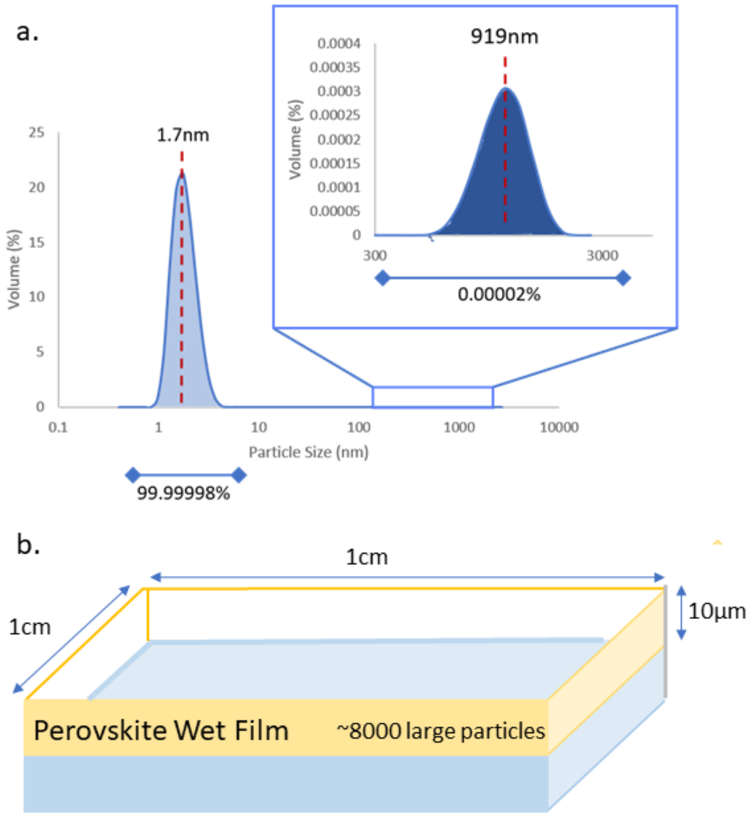

Figure S4. a Volume Percentage Distribution from DLS measurements on TC 2. Integrated percentages measured as the area beneath the curve. **b.** Schematic of the "wet film" used in the DLS

Small-Angle Neutron Scattering

Small angle neutron scattering (SANS) is a powerful technique for probing the average size, shape, distribution, and number of scattering entities in a sample. In a SANS experiment, a collimated beam of neutrons with wave vector $\overrightarrow{k_i}$ is directed towards a sample and neutrons scattered with wave vector $\overrightarrow{k_f}$ at an angle 2θ are collected by a 2D detector, positioned at a distance *L* from the sample (Figure S5). Typically, the measured 2D scattering pattern is radially integrated to generate a 1D intensity profile as a function of the scattering vector $\vec{q}$, defined as the difference between the wave vectors of the incident and scattered neutron beams:

$$\vec{q} = \overrightarrow{k_i} - \overrightarrow{k_f} \qquad \text{Equation S8}$$

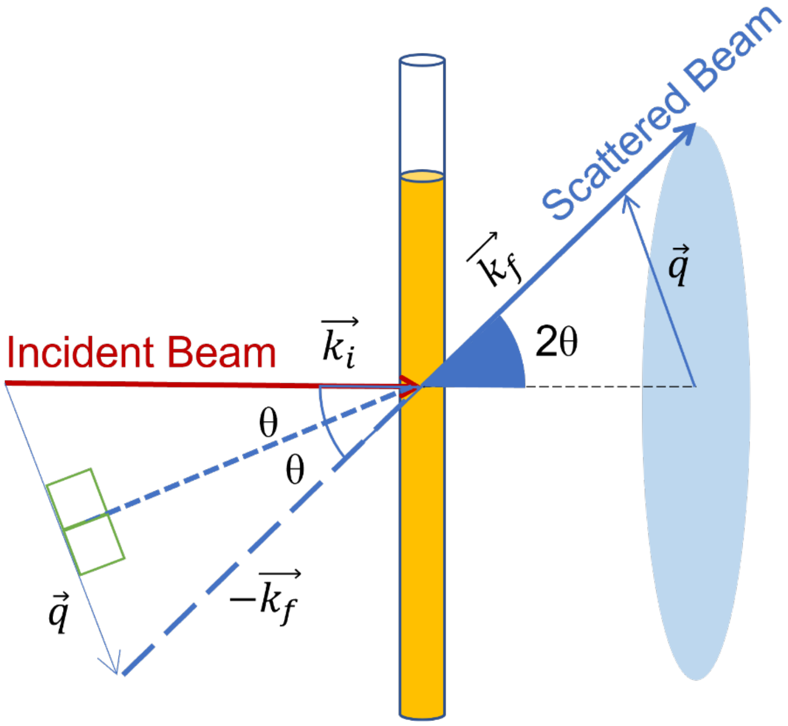

Figure S5. An illustration of the typical transmission geometry used for SANS measurements.

Assuming elastic scattering (i.e., no energy transfer), the magnitude of the incident wave vector is equivalent to the magnitude of the scattered wave vector:

$$|\vec{k_i}| = |\vec{k_f}| = k = \frac{2\pi}{\lambda} \quad \text{Equation S9}$$

where λ is the neutron wavelength calculated from de Broglie's equation ($\lambda = \frac{h}{m_n v}$ where h is Planck's constant, m_n is the mass of the neutron and v is the velocity of the neutron). Using equations (1) and (2), the magnitude of the scattering vector is given by:

$$|\vec{q}| = q = 2\ k sin\theta \qquad \text{Equation S10}$$

$$q = \frac{4\pi sin\theta}{\lambda} \qquad \text{Equation S11}$$

This is the same expression for q used in DLS, except neutron refractive indices are ~1.

The measurable q-range in a SANS experiment is therefore dependent on the wavelength of the neutrons used and the distance L between the detector and the sample. Placing the detector far away from the sample allows small angles to be probed relating to low-q values. Scattering at low-q arises from large length scales d in the system, according to the reciprocal relationship between real-space and q-space:

$$q = \frac{2\pi}{d} \qquad \text{Equation S12}$$

There are many models to describe small angle scattering from different types of nanostructure. Here, the Teubner-Strey model is used to characterise the perovskite precursor solutions. The Teubner-Strey model assumes a two phase system[4]. In this system, the solvent and solutes are the majority and minority phases respectively. The scattering intensity profile over a given q-space can be described using the following equations:

$$I(q) = \frac{8\pi\Phi_a(1-\Phi_a)(\rho_a-\rho_b)^2 c_2/\xi}{a_2 + c_1 q^2 + c_2 q^4} \quad \text{Equation S13}$$

$$a_2 = \left[1 + \left(\frac{2\pi\xi}{d}\right)^2\right]^2 \; and \; c_1 = -2\xi^2\left(\frac{2\pi\xi}{d}\right)^2 + 2\xi^2 \; and \; c_2 = \xi^4$$

Depending on the domain size *d*, correlation length, ξ, volume fraction, Φ_a, and respective scattering length densities, $\rho_{a/b}$. In neutron scattering the phase problem means that *d* can be the domain size or periodicity between particles. We here argue that the *d* from the Teubner-Strey model corresponds to the average domain size of any solute phase and ξ is the average length scale before a phase change. The scattering length densities (SLDs) and volume fraction of the solvent and solute particles can also be extracted using this model. To explore what this could represent in our systems, we refer to Figure S6. Here, we can see that if phase B (the solute) is formed of large aggregates (as seen in S6a), both the domain size *d* and average distance between phases (ξ (= $\Sigma\xi_n$/n)) is larger than for a dispersed solution of nanoparticles (Fig. S5b).

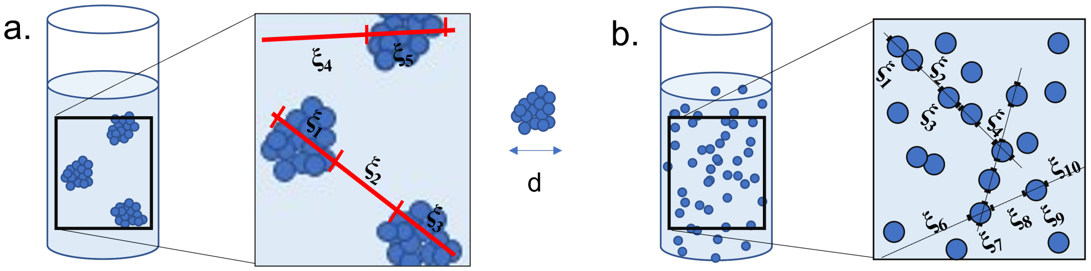

Figure S6. An illustration of large aggregates (**a**) and disperse nanoparticles (**b**) in a solvent material. The solute material representing the minority phase within the Teubner-Strey model is shown in with dark blue circles, with the solvent (i.e., the majority phase) shown in light blue. Insets show the average spacing between "phase changes" for each system, ξ_n, which is averaged to give the correlation length ξ (= Σ ξ_n/n). The domain size of the minority phase is shown for each case as d.

There are several benefits for using neutron scattering to study perovskite solutions compared to conventional small angle X-ray scattering (SAXS) methods. SAXS experiments on perovskite solutions often suffer from low transmission rates as the solutions are very concentrated and Pb has a large scattering cross-section. SANS overcomes this due to the highly penetrative, weakly interacting nature

of neutrons. In contrast to SAXS, which relies on the electromagnetic interaction between the incident X-ray, and differences in electron density of the sample, neutrons are an uncharged probe so only interact with nuclei within the sample. Scattering arises when there is a difference in neutron scattering length densities (SLD) between the atoms, molecules or ions of the solvent and solute in the sample. The neutron SLD of a molecule is equivalent to the summation of nuclear scattering lengths b_i of each constituent atom divided by the molecular volume V_m:

$$SLD = \frac{\sum_{i=1}^{n} b_i}{V_m} \qquad \text{Equation S14}$$

Neutron scattering lengths do not vary linearly across the periodic table as for X-ray scattering lengths - for example hydrogen and deuterium have significantly different neutron SLDs despite having very similar chemical properties. This property was exploited in this work, by preparing samples in deuterated solvents. This provides a large neutron SLD contrast between the solvent and solute in the solution. The calculated SLDs of solvents, perovskite materials and precursor components are shown in Table S1.

**Material**	**Chemical Formula**	**Mass Density (g/cm^3)**	**Neutron SLD ($x10^{-6}$ Å^{-2})***
Deuterated dimethyl sulfoxide (DMSO-d_6)	$(CD_3)_2SO$	1.19	5.28
Deuterated dimethylformamide (DMF-d_7)	$(CD_3)_2NC(O)D$	1.03	6.33
4:1 DMF-d7:DMSO-d6	$(CD_3)_2NC(O)D$: $(CD_3)_2SO$	1.06	6.12
Lead Iodide	PbI_2	6.16	1.61
Lead Bromide	$PbBr_2$	6.66	2.51
Formamidinium Iodide	$HC(NH_2)I$	2.23**	0.93
Methylammonium Bromide	CH_3NH_3Br	1.64**	0.03
Methylammonium Iodide	$CH_3NH_3I_3$	2.00**	-0.07
Caesium Iodide	CsI	4.51	1.11
$MAPbI_3$ perovskite	$CH_3NH_3PbI_3$	4.1-4.2 [5,6]	0.75

$FAPbI_3$ perovskite	$HC(NH_2)PbI_3$	4.1 [5]	1.24
$MAPbBr_3$ perovskite	$CH_3NH_3PbBr_3$	3.81 [6]	1.20

Table S1. A summary of chemical formulae, mass densities and neutron scattering length densities (SLDs) of the solvents, perovskite compositions and respective perovskite precursor materials used in this work. * Scattering length densities determined using [7]. ** Organic material densities estimated using [8].

Film Morphology and Composition

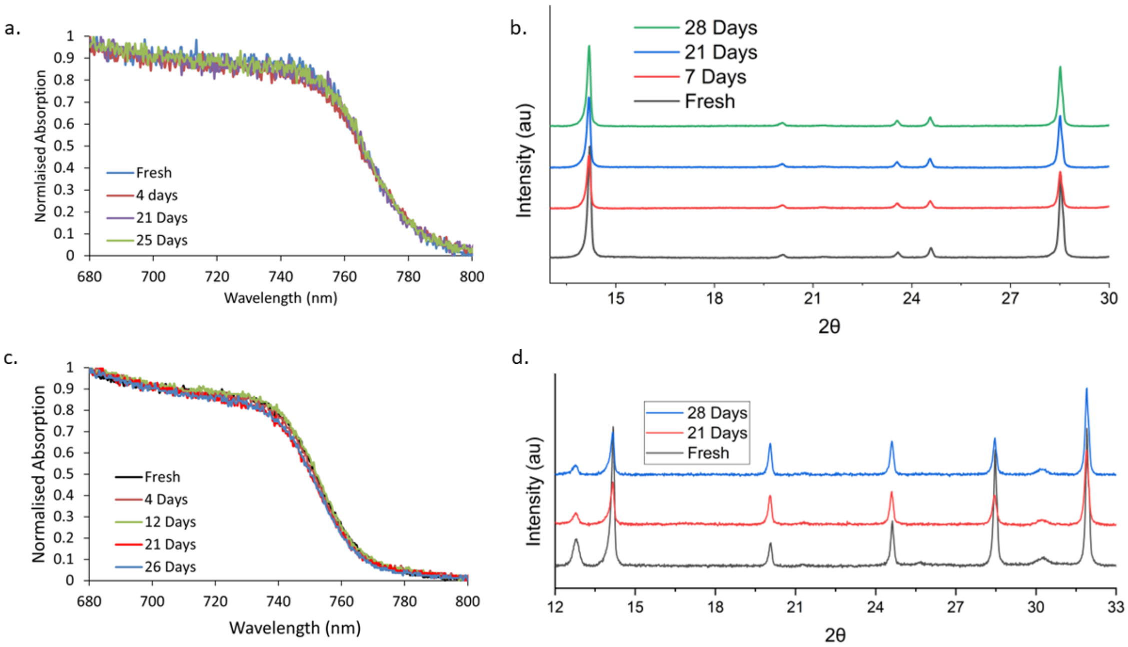

Figure S7. UV-Vis absorption spectra (a) and XRD profiles (b) of films made from a $MAPbI_3$ solution aging over a 1 month period. UV-Vis absorption profiles (c) and XRD profiles (d) of films made from a TC solution aging over a 1 month period.

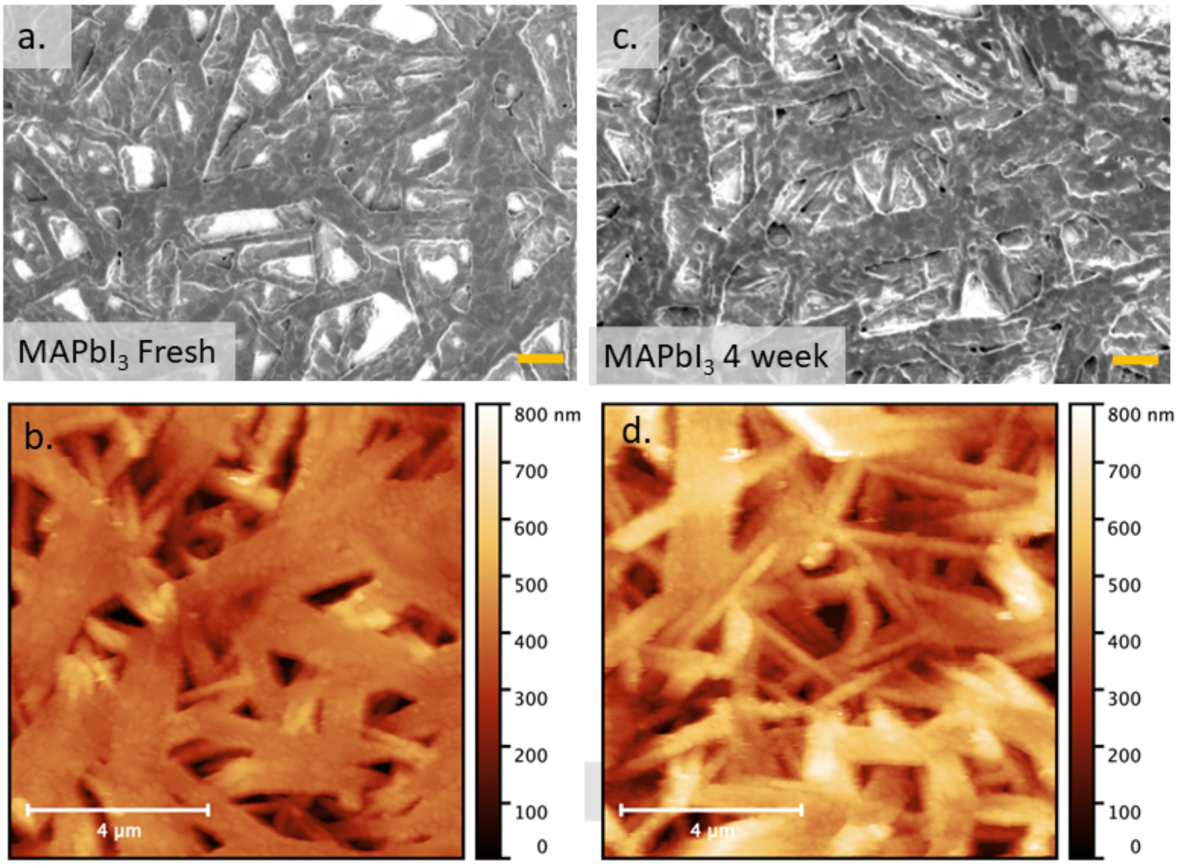

Figure S8. SEM and AFM images of fresh and aged $MAPbI_3$ films. SEM (a) and AFM images (b) of $MAPbI_3$ films made from fresh solution. SEM (c) and AFM images (d) of $MAPbI_3$ films made from solutions aged for 1 month. In the SEM image the scale bars in the bottom right represent 1μm.

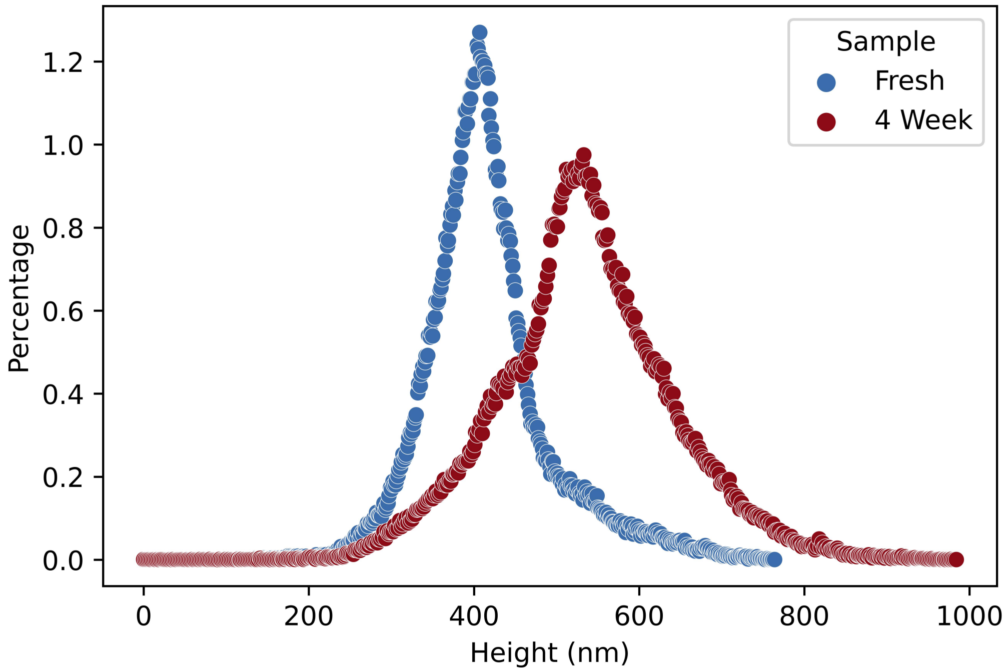

Figure S9. Height histograms taken from AFM images of a film made from a fresh $MAPbI_3$ ink (blue circles) and an aged $MAPbI_3$ ink (red circles).

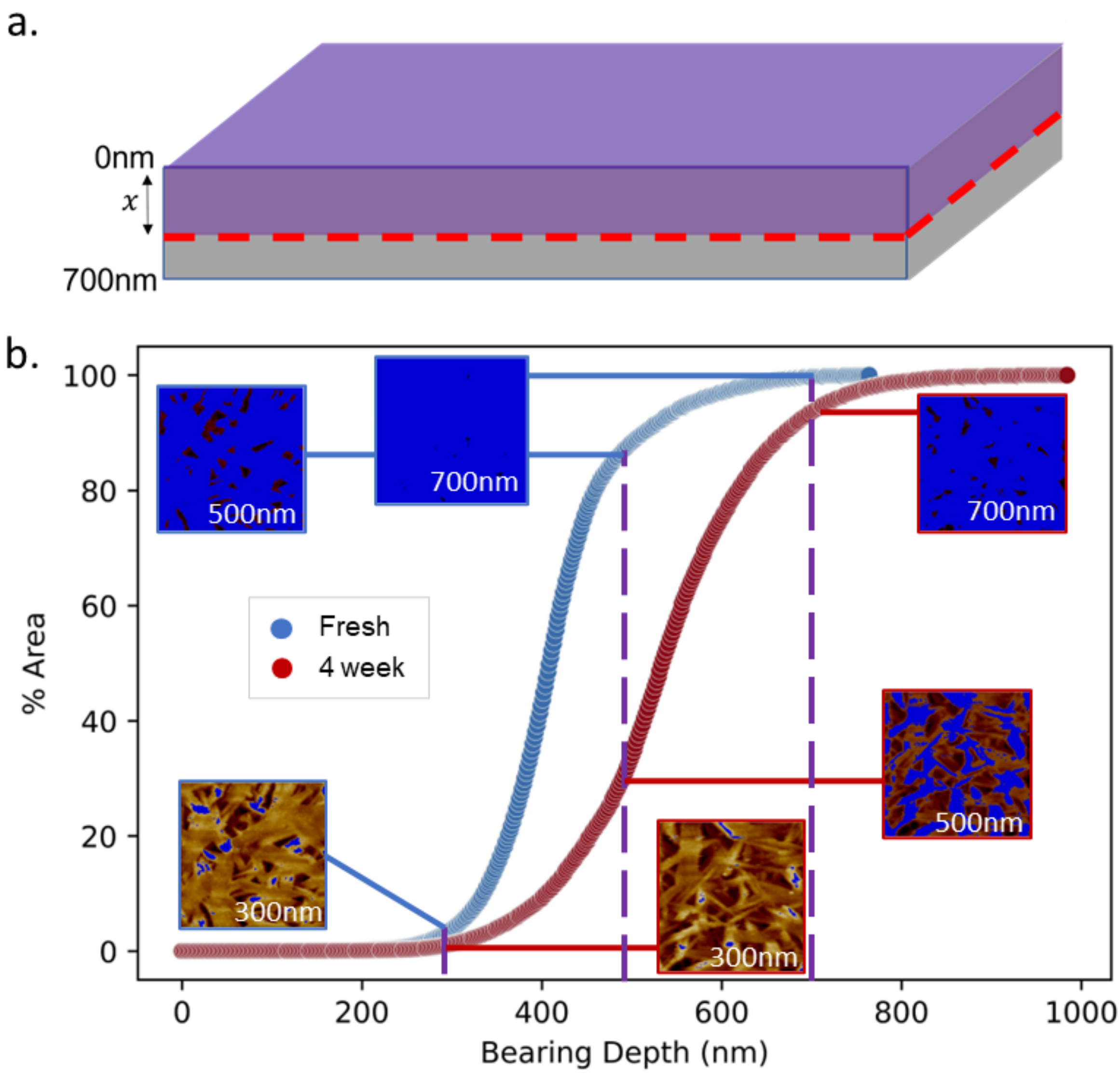

Figure S10. a A diagram showing the penetrating height of bearing analysis. Data given is the percentage area of the film that lies within the bearing depth (marked x) from the top of the film surface. **b.** Bearing analysis data taken from AFM images made from a fresh $MAPbI_3$ ink (blue circles) and an aged $MAPbI_3$ ink (red circles). Insets show % area of the image within the bearing depth from the film surface for fresh $MAPbI_3$ (blue) and aged $MAPbI_3$ (red). This data is calculated from the histograms in Fig. S10.

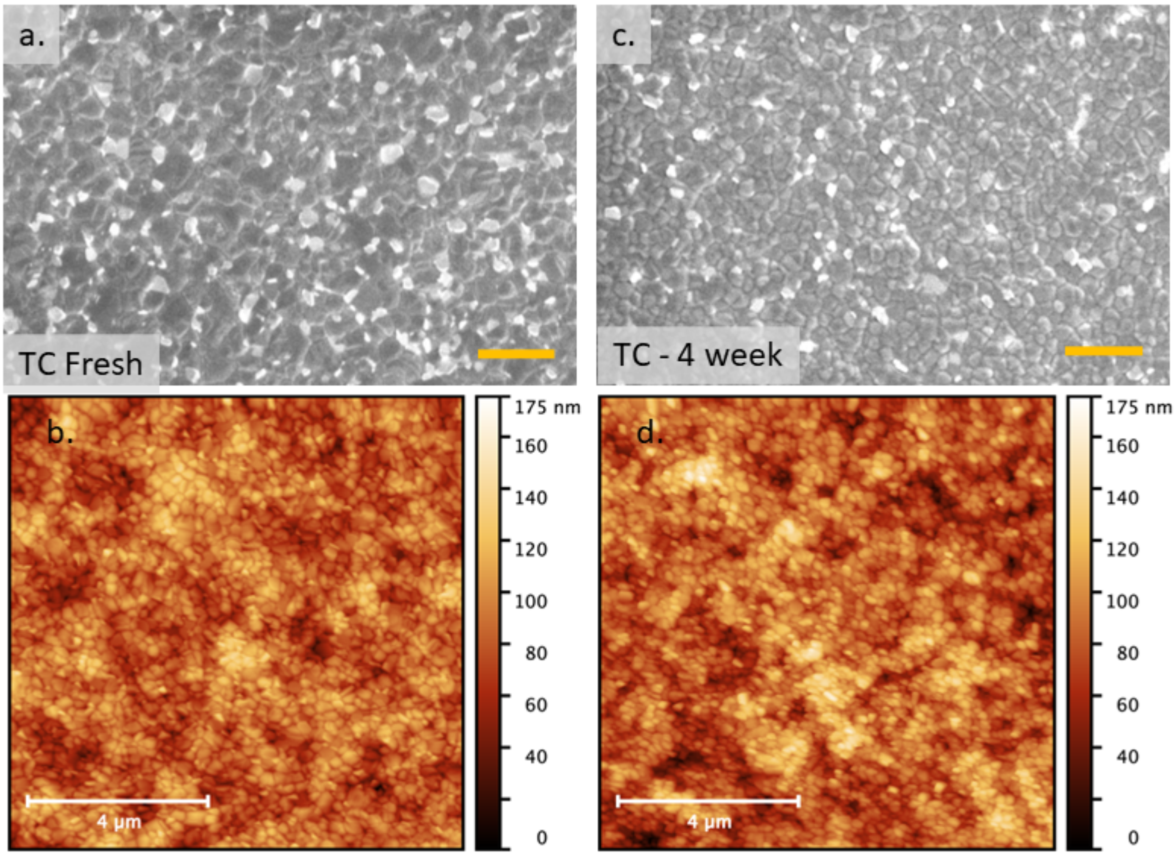

Figure S11. SEM (a) and AFM images (b) of TC films made from fresh solutions. SEM (c) and AFM images (d) of TC films made from aged solutions aged for 1 month. In the SEM image the scale bar in the bottom right represents 1μm.

Spin-Echo Small-Angle Neutron Scattering

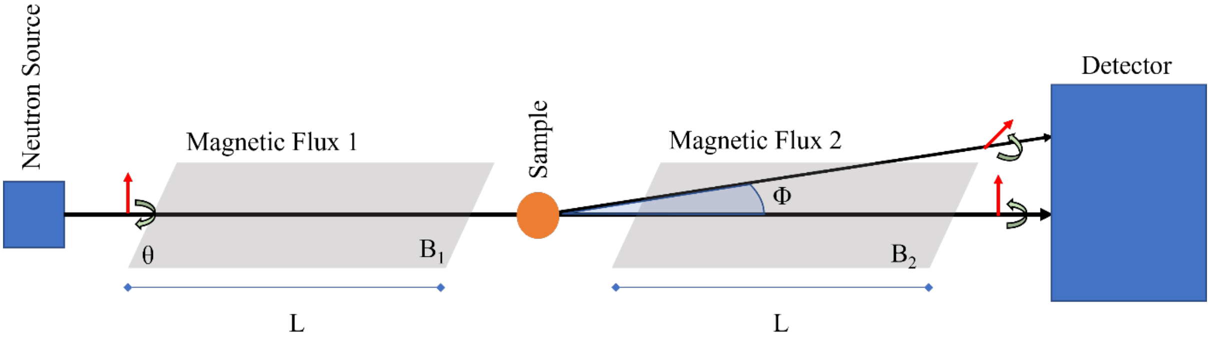

Figure S12. A schematic depicting the basic structure of how a SESANS instrument works.[9]

In a spin-echo SANS (SESANS) instrument the scattering angle is encoded using the neutron spin, this is similar to NMR where ^{1}H nuclear can be measured by exploiting the spin-$1/2$ property of the ^{1}H nuclei and using Larmor precession in magnetic fields. As neutrons are also spin 1/2 particles, we can measure the scattering angle by the use of carefully shaped magnetic fields. The key here is the fact that any accumulated precession is proportional to the strength of the magnetic field and the path traversed by the neutron through the field. In a simplified view if we consider the magnetic field arrangement in Figure S10 with the field strength in B_2 equal and opposite that in B_1 we can see that for any unscattered neutron the field integral comes to zero. However, if the neutron is scattered then the field integral in B_1 is not cancelled out by B_2 and will not come back to the original polarisation (P_0).

By measuring the polarisation of scattered neutrons P_S compared to the polarisation of the unscattered beam P_0,(this allows for the correction of instrumental effects and is measured with no sample). The spin-echo lengths that can be probed using this method are determined using the following equation:

$$z = cBL\lambda^2 \cot\,\theta$$

Where c is a constant, L is the separation between magnets, θ is the inclination of the magnets, B is magnetic field strength and λ is a function of neutron wavelength[10]. Hence with a combination of these parameters we can measure the size of scattering particles in a size range up to 20µm.

The polarisation ($P(z)=P_s(z)/P_0(z)$) of the neutrons as a function of the spin-echo length, z is given by;

$$P(z) = e^{(\Sigma t[(G(z)-1])}$$

Where $\boldsymbol{\Sigma}_t$ is is the fraction of neutrons that are scattered once by a sample of thickness t and G(z) is the projection of the autocorrelation of the density distribution function of the sample[11]. For dilute spheres of radius R (volume fraction (Φ) >5%) this quantity $\boldsymbol{\Sigma}_t$ is given by;

$$\boldsymbol{\Sigma}_t = 3/2Rt\,\Phi(1-\Phi)\,(\rho_a - \rho_b)^2\lambda^2$$

Where t is the sample thickness and λ the neutron wavelength, the scattering length density contrast $(\rho_a - \rho_b)^2$, the same as in the SANS case. Hence for any known particle size and contrast we can determine the volume fraction Φ.

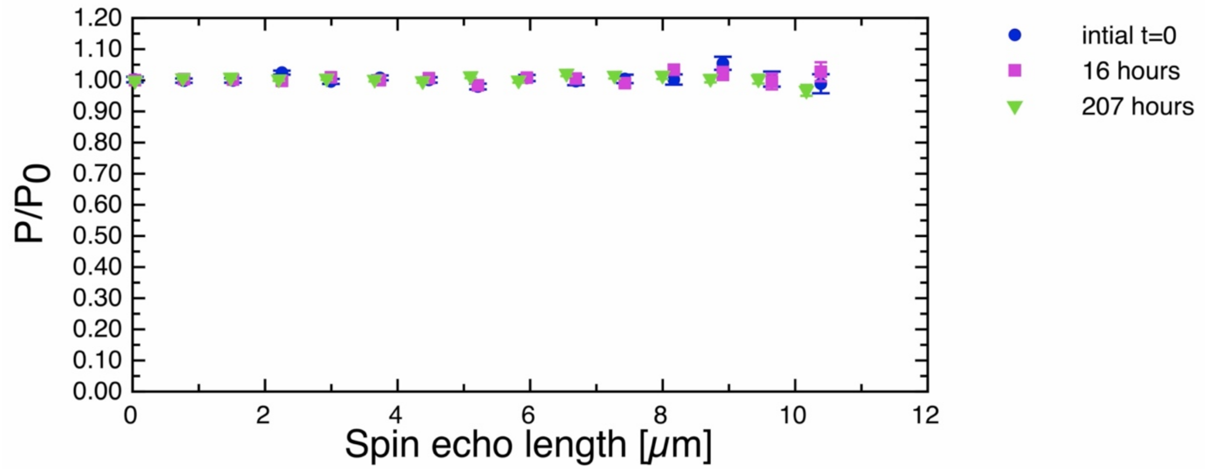

Figure S13. SESANS data showing the ratio of P/P_0 for the precursor ink solution of $CsFAMAPb(Br_xI_{x-1})_3$ showing the complete lack of depolarisation. These data were collected continuously for a period of 9 days.

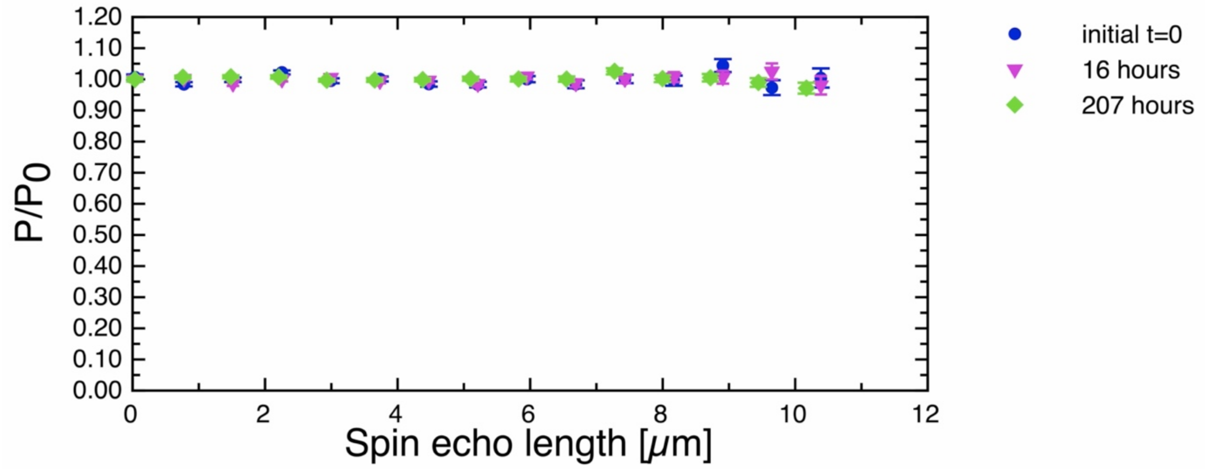

Figure S14. SESANS data showing the ratio of P/P_0 for $MAPbI_3$ again also showing a lack of depolarisation. These data were also collected continuously for a period of 9 days.

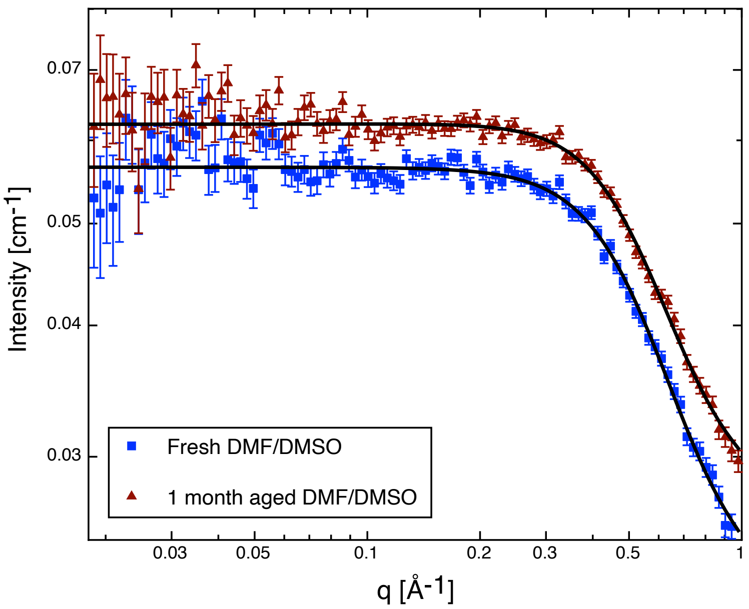

Figure S15. SANS data for $MAPbI_3$ in the same solvent mixture (DMF/DMSO) as that used for the $CsFAMAPb(Br_xI_{x-1})_3$ solutions.

References

(1) MALVERN. Zetasizer Nano Series. *Malvern Instruments Ltd* **2014**, 20. https://doi.org/10.1016/0141-0229(93)90071-9.

(2) Stetefeld, J.; McKenna, S. A.; Patel, T. R. Dynamic Light Scattering: A Practical Guide and Applications in Biomedical Sciences. *Biophys. Rev.* **2016**, *8* (4), 409–427. https://doi.org/10.1007/s12551-016-0218-6.

(3) Malvern Instruments. *Technical Note: Dynamic Light Scattering*; 2010. http://www.aim.env.uea.ac.uk/aim/density/density.php (accessed 22-02-22)

(4) Teubner Strey Model, *SASView Version 4.2.2*. https://www.sasview.org/docs/user/models/teubner_strey.html (accessed 2022 -03 -22).

(5) Stoumpos, C. C.; Malliakas, C. D.; Kanatzidis, M. G. Semiconducting Tin and Lead Iodide Perovskites with Organic Cations: Phase Transitions, High Mobilities, and near-Infrared Photoluminescent Properties. *Inorg. Chem.* **2013**, *52* (15), 9019–9038. https://doi.org/10.1021/ic401215x.

(6) Jaffe, A.; Lin, Y.; Beavers, C. M.; Voss, J.; Mao, W. L.; Karunadasa, H. I. High-Pressure Single-Crystal Structures of 3D Lead-Halide Hybrid Perovskites and Pressure Effects on Their Electronic and Optical Properties. *ACS Cent. Sci.* **2016**, *2* (4), 201–209.

https://doi.org/10.1021/acscentsci.6b00055.

(7) Al., M. D. et. *SASView Version 4.2.2*. https://doi.org/10.5281/zenodo.2652478.

(8) Densities of Pure Liquid Organic Compounds http://www.aim.env.uea.ac.uk/aim/density/density.php (accessed 2022 -03 -22).

(9) Rekveldt, M. T.; Plomp, J.; Bouwman, W. G.; Kraan, W. H.; Grigoriev, S.; Blaauw, M. Spin-Echo Small Angle Neutron Scattering in Delft. *Rev. Sci. Instrum.* **2005**, *76* (3), 033901. https://doi.org/10.1063/1.1858579.

(10) Bernardo, G.; Melle-Franco, M.; Washington, A. L.; Dalgliesh, R. M.; Li, F.; Mendes, A.; Parnell, S. R. Different Agglomeration Properties of PC 61 BM and PC 71 BM in Photovoltaic Inks – a Spin-Echo SANS Study. *RSC Adv.* **2020**, *10* (8), 4512–4520. https://doi.org/10.1039/C9RA08019H.

(11) Mulder, M.; Li, X. X.; Nazim, M. M.; Dalgliesh, R. M.; Tian, B.; Buijse, M.; van Wunnik, J.; Bouwman, W. G. Systematically Quantifying Oil–Water Microemulsion Structures Using (Spin-Echo) Small Angle Neutron Scattering. *Colloids Surfaces A Physicochem. Eng. Asp.* **2019**, *575* (April), 166–175. https://doi.org/10.1016/j.colsurfa.2019.04.045.
